# First Detection and Molecular Identification of *Borrelia garinii* Spirochete from *Ixodes ovatus* Tick Ectoparasitized on Stray Cat in Taiwan

**DOI:** 10.1371/journal.pone.0110599

**Published:** 2014-10-24

**Authors:** Li-Lian Chao, Li-Ling Liu, Tsung-Yu Ho, Chien-Ming Shih

**Affiliations:** 1 Graduate Institute of Pathology and Parasitology, Department of Parasitology and Tropical Medicine, National Defense Medical Center, Taipei, Taiwan, R.O.C.; 2 Graduate Institute of Medicine, College of Medicine, Kaohsiung Medical University, Kaohsiung, Taiwan, R.O.C.; 3 Center for Infectious Disease and Cancer Research, Kaohsiung Medical University, Kaohsiung, Taiwan, R.O.C.; University of North Dakota School of Medicine and Health Sciences, United States of America

## Abstract

*Borrelia garinii* spirochete was detected for the first time in *Ixodes ovatus* tick ectoparasitized on stray cat in Taiwan. The genetic identity of this detected spirochete was determined by analyzing the gene sequence amplified by genospecies-specific polymerase chain reaction assays based on the 5S–23S intergenic spacer amplicon (*rrf*-*rrl*) and outer surface protein A (*ospA*) genes of *B. burgdorferi* sensu lato. Phylogenetic relationships were analyzed by comparing the sequences of *rrf*-*rrl* and *ospA* genes obtained from 27 strains of *Borrelia* spirochetes representing six genospecies of *Borrelia*. Seven major clades can be easily distinguished by neighbour-joining analysis and were congruent by maximum-parsimony method. Phylogenetic analysis based on *rrf-rrl* gene revealed that this detected spirochete (strain IO-TP-TW) was genetically affiliated to the same clade with a high homogeneous sequences (96.7 to 98.1% similarity) within the genospecies of *B. garinii* and can be discriminated from other genospecies of *Borrelia* spirochetes. Interspecies analysis based on the genetic distance values indicates a lower level (<0.022) of genetic divergence (GD) within the genospecies of *B. garinii*, and strain IO-TP-TW was genetically more distant ( >0.113) to the strains identified in *I*. *ovatus* collected from Japan and China. Intraspecies analysis also reveals a higher homogeneity (GD<0.005) between tick (strain IO-TP-TW) and human (strain Bg-PP-TW1) isolates of *B. garinii* in Taiwan. This study provides the first evidence of *B. garinii* isolated and identified in an *I*. *ovatus* tick in Asia, and the higher homogeneity of *B. garinii* between tick and human strain may imply the risk of human infection by *I*. *ovatus* bite.

## Introduction

The causative agent for Lyme disease, *Borrelia burgdorferi* sensu lato, was firstly identified within the gut of *Ixodes* tick [1] and the spirochete species can be classified into at least thirteen genospecies based on their genetic differences [2]–[5]. The tick species of *Ixodes ricinus* complex serve as the main vectors for transmission and perpetuation of *B. burgdorferi* spirochetes through a natural cycle between vector ticks and rodent hosts in North America and Europe [6], [7]. Although the tick species of *I. persulcatus* has been recognized as the principle vector for the transmission of *B. burgdorferi* spirochetes in Northeast Asia, including the northeastern regions of China, Korea, and Japan [8]–[11], the hard ticks of *I. granulatus*, *Haemaphysalis longicornis*, and *H. bispinosa* were incriminated as the main vectors for the transmission of *B. burgdorferi* spirochetes in the southwestern regions of China and Taiwan [12]–[14].

The abundance and geographical distribution of the tick species of *I. ovatus* has been recorded from various countries in Southeast Asia, Taiwan, Japan, Korea and China [15]–[17]. Human biting activities have also been observed in the countries of Tibet, Burma, Nepel, Japan and China [18]. In Japan, this tick species was responsible for the abundance of tick bites on humans. However, no human cases of Lyme disease transmitted by *I. ovatus* have been confirmed in Japan [19]–[21]. In addition, the *Borrelia* spirochetes isolated from *I. ovatus* ticks are thought to be a variant strain with low virulence to humans, and were further identified as a new strain of *B. japonica* and *B. sinica* in Japan and China, respectively [22], [23]. In our previous investigations, Lyme disease spirochetes (*B. burgdorferi* sensu lato) were isolated and identified from rodent hosts and human skin specimens in Taiwan [24]–[27]. Although *B. burgdorferi* sensu stricto and *B. valaisiana* have been detected in *I. granulatus* ticks [28], [29], spirochetal isolation from *I. ovatus* tick infested on cat has never been reported in Taiwan.

Genomic analysis among *Borrelia* isolates by sequence similarity of a specific target gene has proven useful for the species identification and genomic typing of *Borrelia* spirochetes isolated from different biological and geographical sources [30]–[32]. Outer surface protein (osp) genes in all *Borrelia* isolates belonging to the major genospecies of *B. burgdorferi* sensu lato were verified and described [33], [34]. The 5S (*rrf*) –23S (*rrl*) intergenic spacer amplicon gene is unique and highly conserved in *B. burgdorferi* sensu lato [35], [36]. The genetic diversity of these genes are useful for distinguishing the genetic heterogeneity among different *Borrelia* isolates [37]–[41]. Indeed, the genetic identity of *Borrelia* isolates had been clarified by analyzing the sequence homology of the 5S (*rrf*) –23S (*rrl*) intergenic spacer amplicon and ospA genes of *Borrelia* spirochetes isolated from various biological sources [25]–[28], [31], [42], [43].

It is assumed that different genospecies of *B. burgdorferi* sensu lato are distributed unevently throughout the world and are associated with distinct ecologic features [44]. It may be that the genospecies of *Borrelia* spirochete existed in *I. ovatus* tick of Taiwan is distinct from the *Borrelia* spirochetes within *I. ovatus* ticks identified in Japan and China. Thus, the objectives of the present study intend to clarify the genospecies of *Borrelia* spirochete isolated from the *I. ovatus* tick collected from cat of Taiwan, by analyzing the sequence similarity of the PCR-amplified 5S (*rrf*) –23S (*rrl*) intergenic spacer amplicon and ospA genes. In addition, the phylogenetic relationships of the detected spirochete was compared with other *Borrelia* species documented in GenBank as well as the *Borrelia* spirochetes identified from various biological sources, including human, rodent and tick in Taiwan.

## Materials and Methods

### Collection and identification of tick specimen

All specimens of adult ticks of *I. ovatus* were removed from stray cats captured at various residential sites of Neihu district in Taipei City of northern Taiwan (25^o^5′23.3″N 121^o^35′33.8″E). A total of fifteen adult ticks (10 female and 5 male) were collected and all these ticks were subsequently stored in separate mesh-covered and plaster-bottomed vials. Adult ticks of male and female *I. ovatus* were identified to species level on the basis of their morphological characteristics, as described previously [14], [45]. Ultrastructural observations by stereomicroscope were used to delineate the morphological features of adult *I. ovatus* ticks in Taiwan. Briefly, tick specimens were cleaned by sonication in 70% ethanol solution for 5-10 min and then washed twice in sterile distilled water. Afterwards, each tick specimen was placed on a glass slide and photographed using a stereomicroscope (SMZ 1500, Nikon, Tokyo, Japan) equipped with a fiber lamp. The external features of the male and female *I. ovatus* ticks were recorded for species identification.

### Isolation and purification of *Borrelia* spirochetes

For the isolation of spirochetes, tick specimens were cleaned by sonication for 3–5 min in 75% ethanol solution and then washed twice in sterile distilled water. Afterwards, specimen of each individual adult tick was dissected into pieces and inoculated into a culture tube containing BSK-H medium (B3528; Sigma Co., St. Louis, MO, USA) supplemented with 6% rabbit serum (R7136; Sigma), as described previously [46]. After incubation at 34°C in a humidified incubator with 5% CO_2_, tick cultures were examined weekly for 8 weeks for the evidence of spirochetes by dark-field microscope (E400, Nikon) equipped with a digital camera. For the purification of cultured spirochetes, spirochete-positive culture with other contamination was transferred to new culture tubes by serial dilution and were further filtered with a 0.45-µm pore size syringe filter (Sartorius, Gottingen, Germany), as described previously [24], [47]. After further incubation for two weeks, the pure spirochete-positive cultures were stored in a deep freezer (−80°C) until further analysis.

### DNA extraction from spirochete culture

Total genomic DNA was extracted from individual spirochete-positive culture with the DNeasy Tissue Kit (catalog No. 69506; Qiagen, Taipei, Taiwan) and used as a template for PCR amplification. Briefly, individual positive-culture medium together with dissected tick tissue was placed in a microcentrifuge tube and centrifuged for 20 min at 12000×g to pellet the spirochetes. After removing the supernatant, the microcentrifuge tube was filled with 180 µl lysing buffer solution and was further processed with the DNeasy Tissue Kit, as per manufacturer's instruction. After filtration, the eluate was collected and the DNA concentration was determined spectrophotometrically with a DNA calculator (Gene-Quant II; Pharmacia Biotech, Uppsala, Sweden).

### DNA amplification by *Borrelia*-specific polymerase chain reaction (PCR)

DNA sample extracted from the spirochete culture was used as a template for PCR amplification. A nested PCR was performed with primers designed to amplify the variable spacer region of *rrf-rrl* gene between two conserved duplicate structures. A specific primer set corresponding to the 3′ end of the 5S rRNA (*rrf*) (5′-CGACCTTCTTCGCCTTAAAGC-3′) and the 5′ end of the 23S rRNA (*rrl*) (5′-TAAGCTGACTAATACTAATTACCC-3′) was designed and applied for the primary amplification, as described previously [36]. In the nested PCR, a primer set of primer 1 (5′-CTGCGAGTTCGCGGGAGA-3′) and primer 2 (5′-TCCTAGGCATTCACCATA-3′) was used and expected to yield a 226-266 bp fragment depending on the *Borrelia* strain, as described previously [48]. For *ospA* gene, specific primer sets of N1 (5′-GAGCTTAAAGGAACTTCTGATAA-3′)/C1 (5′-GTATTGTTGTACTGTAATTGT-3′) and N2 (5′-ATGGATCTGGAGTACTTGAA-3′)/C2 (5′-CTTAAAGTAACAGTTCCTTCT-3′) were designed for primary and secondary amplification, as described previously [49]. All PCR reagents and Taq polymerase were obtained and used as recommended by the manufacturer (Takara Shuzo Co., Ltd., Japan). Briefly, a total of 0.2 µmol of the appropriate primer set and various amounts of template DNA were used in each 50-µl reaction mixture. PCR amplification was performed with a Perkin-Elmer Cetus Thermocycler (GeneAmp System 9700; Taipei, Taiwan). The primary amplification for *rrf-rrl* gene included 2 min denaturation at 96°C followed by 30 cycles of the following conditions: denaturation at 94°C for 30 s, annealing at 55°C for 30 s, and extension at 72°C for 40 s, and the nested amplification was performed under the same conditions except the annealing at 59°C for 30 s. For *ospA* gene, the primary amplification included 1 min denaturation at 95°C followed by 35 cycles of the following conditions: denaturation at 95°C for 45 s, annealing at 45°C for 45 s, and extension st 72°C for 60 s, and the nested amplification was performed under the same conditions except the annealing at 50°C for 45 s. Thereafter, PCR-amplified DNA product was electrophoresed on 2% agarose gel in Tris-borate-EDTA (TBE) buffer and was visualized under ultraviolet (UV) light after staining with ethidium bromide. A DNA ladder (1-kb plus, catalog No. 10787–018; Invitrogen, Taipei, Taiwan) was used as the standard marker for comparison. A negative control of distilled water was included in parallel with each amplification.

### Sequence alignment and phylogenetic analysis

After purification (QIAquick PCR Purification Kit, catalog No. 28104), sequencing reaction was performed with 25 cycles under the same conditions and same primer set (primer 1 and primer 2) of nested amplification by dye-deoxy terminator reaction method using the Big Dye Terminator Cycle Sequencing Kit in an ABI Prism 377–96 DNA Sequencer (Applied Biosystems, Foster City, CA, USA). The resulting sequence was initially edited by BioEdit software (V5.3) and aligned with the CLUSTAL W software [50]. Thereafter, the aligned sequence was further analyzed by comparing with other *Borrelia* sequences based on the type-strain of different genospecies and different geographical/biological origin of *Borrelia* spirochetes that available in GenBank. Phylogenetic analysis was performed by neighbour-joining (NJ) compared with maximum parsimony (MP) methods to estimate the phylogeny of the entire alignment using MEGA 4.0 software package [51]. A similarity matrix was also constructed using the DNASTAR program (Lasergene, version 8.0). The genetic distance values of inter- and intra-species variations of *Borrelia* spirochetes were also analyzed by the Kimura two-parameter model [52]. All phylogenetic trees were constructed and performed with 1000 bootstrap replications to evaluate the reliability of the construction, as described previously [53].

### Nucleotide sequence accession numbers

The nucleotide sequences of PCR-amplified 5S (*rrf*) –23S (*rrl*) intergenic spacer amplicon and outer surface protein A (*ospA*) genes from *Borrelia* spirochete (strain IO-TP-TW) determined in this study have been registered and assigned the GenBank accession number of KJ577538 and KM397123, respectively. In the phylogenetic analysis, the nucleotide sequences of the 5S (*rrf*) –23S (*rrl*) intergenic spacer amplicon and *ospA* genes from other 27 strains of *Borrelia* spirochetes were included for comparison and their GenBank accession numbers are shown in Table 1.

**Table 1:** Genospecies and strains of *Borrelia* spirochetes used for analysis in this study.

Genospecies and strain	Origin of *Borrelia* strain		Genbank accession number^a^	
	Biological	Geographic	*rrf*-*rrl* gene	*OspA* gene
*B. burgdorferi* sensu stricto				
B31	*Ixodes scapularis*	USA	L30127	AY030279
IP1	Human CSF	France	AF090977	
Twkm5	*Rattus norvegicus*	Taiwan	AY032908	AF369941
KH-13	*I. granulatus*	Taiwan	JF970245	
Bbss-MP-TW	Human skin	Taiwan	GQ499848	
*B. garinii*				
20047	*I. ricinus*	France	L30119	
NT29	*I. persulcatus*	Japan	L30130	
CT7p	*I. persulcatus*	China	AB035963	
Tr309	*I. ricinus*	Turkey	AB035963	
Bg-PP-TW1	Human skin	Taiwan	JX649205	
** IO-TP-TW**	***I. ovatus***	**Taiwan**	**KJ577538**	**KM397123**
SKT-7.1	*I. ricinus*	Slovakia		GU906888
Khab457	*I. persulcatus*	Russia		AY260455
Tom 5202	*I. persulcatus*	Russia		DQ479285
PBi	Human CSF	Germany		X80257
PTrob	Human skin	Slovenia		X80186
*B. valaisiana*				
VS116	*I. ricinus*	Switzerland	L30134	Y10840
CKA2a	*Apodemus agrarius*	China	AB022124	
M49	*I. ricinus*	Netherland		AF095945
*B. afzelii*				
VS461	*I. ricinus*	Switzerland	L30135	Z29087
Khab	*I. persulcatus*	Russia		AY502599
J1	*I. persulcatus*	Japan	L30129	
Ba-PP-TW1	Human skin	Taiwan	JX649207	
Ba-MP-TW	Human skin	Taiwan	GQ499849	
*B. japonica*				
Cow611C	*I. ovatus*	Japan	L30125	Y10890
HO14	*I. ovatus*	Japan	L30128	Y10893
*B. sinica*				
CWO1	*I. ovatus*	China	AB022130	
NNIo	*I. ovatus*	Nepel	AB100435	

a GenBank accession numbers for *rrf*-*rrl* (**KJ577538**) and *OspA* (**KM397123**) were submitted by this study.

### Ethics Statement

This study was approved, and carried out within strict accordance of the guidelines, by the Ethical Committee of the Institutional Review Board of National Defense Medical Center (IACUC-09-183). All animal process was operated by the Taipei City Animal Protection Office and was adhered to the Animal Protection Law of the Taipei City Government. This study did not involve endangered species and no specific permissions were required for these collections.

## Results

### Species identification of tick specimen

**Figure 1:**
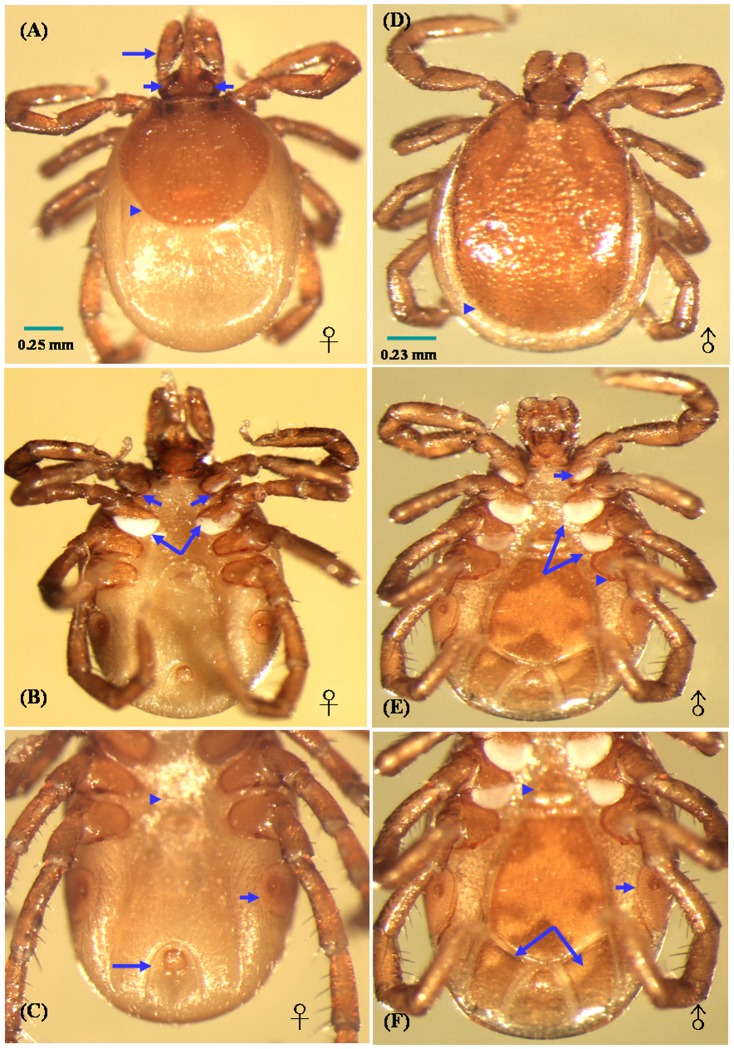
Light micrographs of female (A, B, and C) and male (D, E, and F) *Ixodes ovatus* ectoparasitized on cat in Taiwan. **A**, Dorsal view of female tick showing one pair of long palp (*long arrow*), oval porose areas (*short arrows*) situated at the subpentagonal basis capituli and the oval-shaped scutum covered on a half of abdomen (*arrowhead*); **B**, Ventral view of female tick showing a special transparent membrane-like structure (white sheet) covered on a third of coxae I (*short arrows*) and two third of coxae II (*long arrows*); **C**, The genital aperture (*arrowhead*) is situated at a level between coxae III and IV. Laterally, the rounded spiracular plates (*short arrow*) contain large maculae. The oval-shaped anal groove (*long arrow*) encircled around the anus with a posterior opening was situated at the end of abdomen; **D**, Dorsal view of male tick showing the oval-shaped scutum covered on full-abdomen (*arrowhead*); **E**, Ventrally, coxae IV of male tick possess short, distinct external spurs (*arrowhead*), and coxae I–III was characterized with a special transparent membrane-like structure (white sheet) covered on a third of coxae I (*short arrow*), two third of coxae II and a half of coxae III (*long arrows*); **F**, The genital aperture (*arrowhead*) is situated at level between coxae III. Laterally, the oval-shaped spiracular plates (*short arrow*) contain small maculae. The adenal plates (*long arrows*) paralleled with the anus were only observed on male tick.

Ultrastructural features of adult female *I. ovatus* include four pairs of leg, one pair of long palp (long arrow in Fig. 1A) and oval porose areas (short arrows in Fig. 1A) situated on the subpentagonal basis capituli. The oval-shaped scutum covered on a half of abdomen (arrowhead in Fig. 1A). Ventrally, coxae I possess short, distinct internal spurs and was characterized with a special transparent membrane-like structure (white sheet) covered on a third of coxae I (short arrows in Fig. 1B) and two third of coxae II (long arrows in Fig. 1B). The genital aperture (arrowhead in Fig. 1C) is situated at a level between coxae III and IV. Laterally, the rounded spiracular plates (short arrow in Fig. 1C) contain large maculae. The oval-shaped anal groove (long arrow in Fig. 1C) is curved and situated posterior to the anus, and the posterior body margin bears no festoons. Dorsal view of adult male *I. ovatus* was characterized with a subpentagonal basis capituli and oval-shaped scutum covered on full-abdomen (arrowhead in Fig. 1D). Ventrally, coxae IV possess short, distinct external spurs (arrowhead in Fig. 1E), and coxae I to III was characterized with a special transparent membrane-like structure (white sheet) covered on a third of coxae I (short arrow in Fig. 1E), two third of coxae II and a half of coxae III (long arrows in Fig. 1E), respectively. The genital aperture (arrowhead in Fig. 1F) is situated at level between coxae III. Laterally, the oval-shaped spiracular plates (short arrow in Fig. 1F) contain small maculae. One pair of adanal plate was observed in parallel with the anus (long arrows in Fig. 1F).

### Cultivation of spirochetes from *I. ovatus* tick

**Figure 2:**
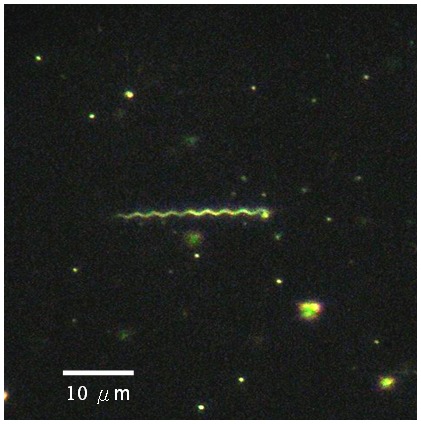
Light micrograph of pure isolate of *Borrelia* spirochete observed in culture medium at 3 to 7 days after filtrated passage.

Cultivation of spirochetes from tick specimen demonstrates that *B. burgdorferi*-like spirochetes were only detected in tissue culture of one tick at 11 days after initial inoculation. Purification of cultivable spirochetes was performed by serial dilution of filtrated cultures and pure isolate of *B. burgdorferi*-like spirochetes was also observed in culture medium at 3 to 7 days after filtrated passage (Fig. 2). To obtain sufficient quantity of cultivable spirochetes, all positive cultures were allowed to grow in BSK-H medium for another 2 weeks.

### Genetic and phylogenetic analysis of cultivable spirochete

**Figure 3:**
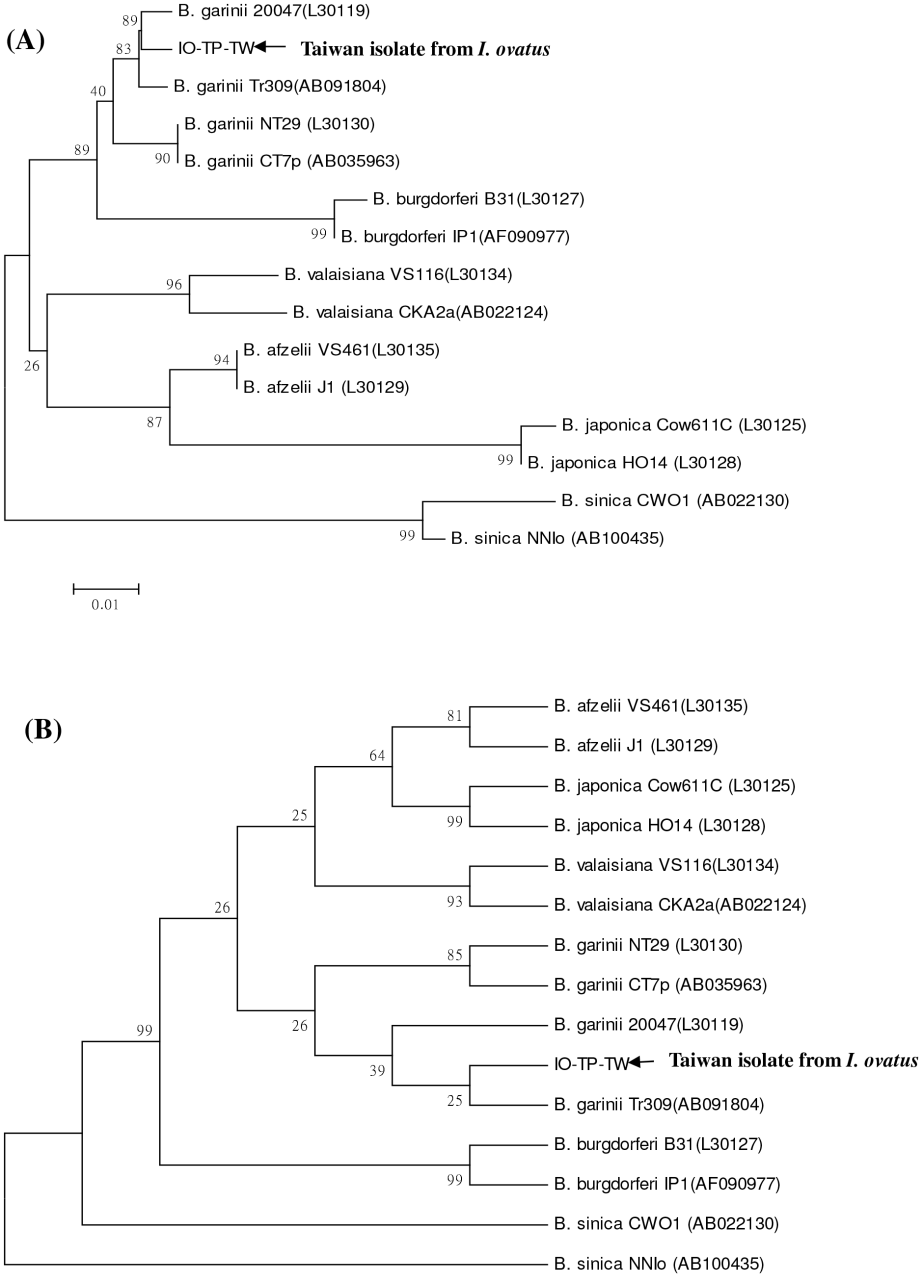
Phylogenetic relationships based on the 5S (*rrf*) –23S (*rrl*) rRNA gene sequences between *Borrelia* spirochete isolated from *I. ovatus* tick (strain IO-TP-TW) in Taiwan and 14 other strains belonging to six genospecies of *Borrelia* spirochetes. The trees were constructed and analyzed by (**A**) neighbour-joining and (**B**) maximum parsimony methods using 1000 bootstraps replicates. Numbers at the nodes indicate the percentages of reliability of each branch of the tree. Branch lengths are drawn proportional to the estimated sequence divergence.

**Figure 4:**
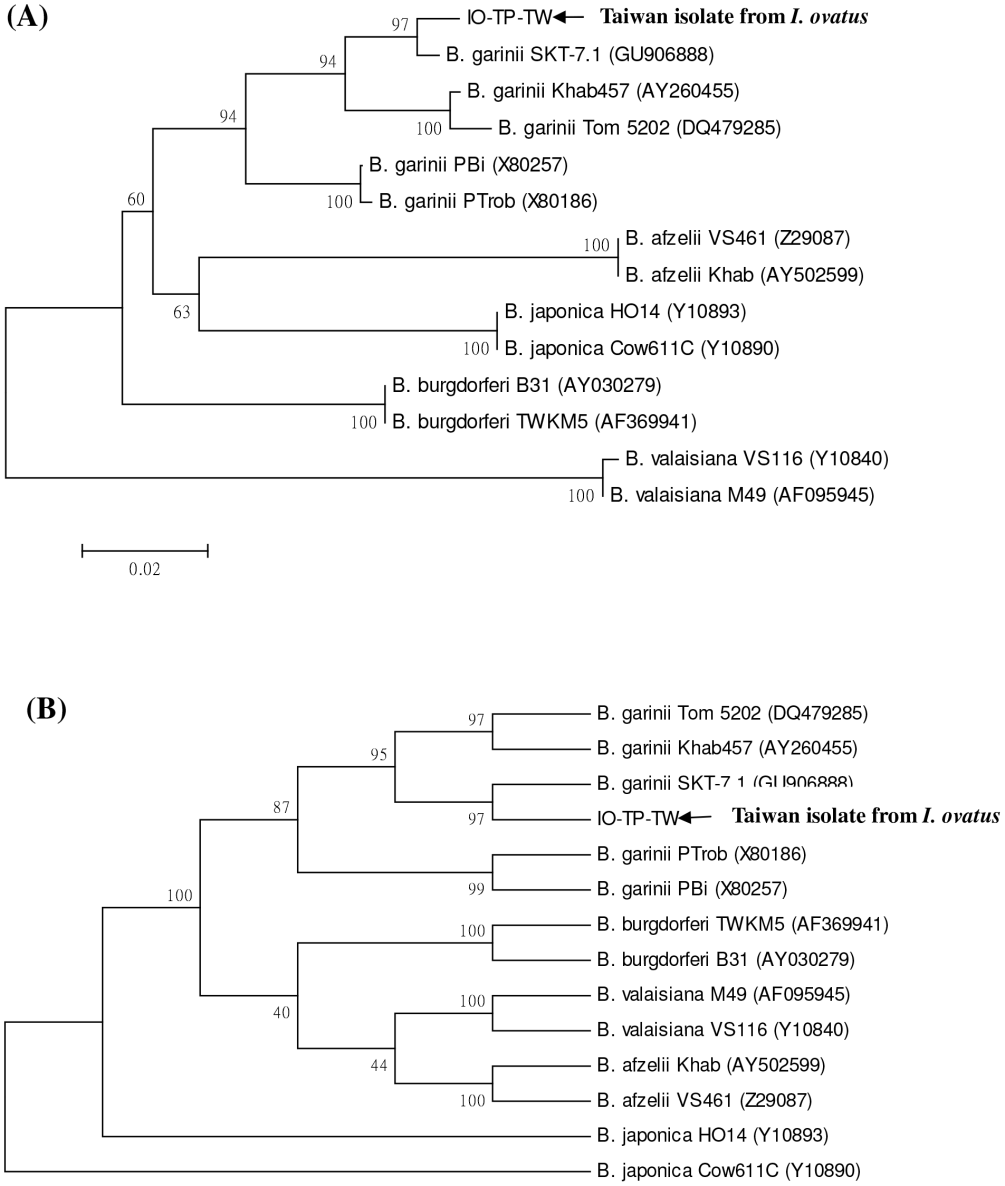
Phylogenetic relationships based on the outer surface protein A (OspA) gene sequences between *Borrelia* spirochete isolated from *I. ovatus* tick (strain IO-TP-TW) in Taiwan and 13 other strains belonging to six genospecies of *Borrelia* spirochetes. The trees were constructed and analyzed by (**A**) neighbour-joining and (**B**) maximum parsimony methods using 1000 bootstraps replicates. Numbers at the nodes indicate the percentages of reliability of each branch of the tree. Branch lengths are drawn proportional to the estimated sequence divergence.

**Figure 5:**
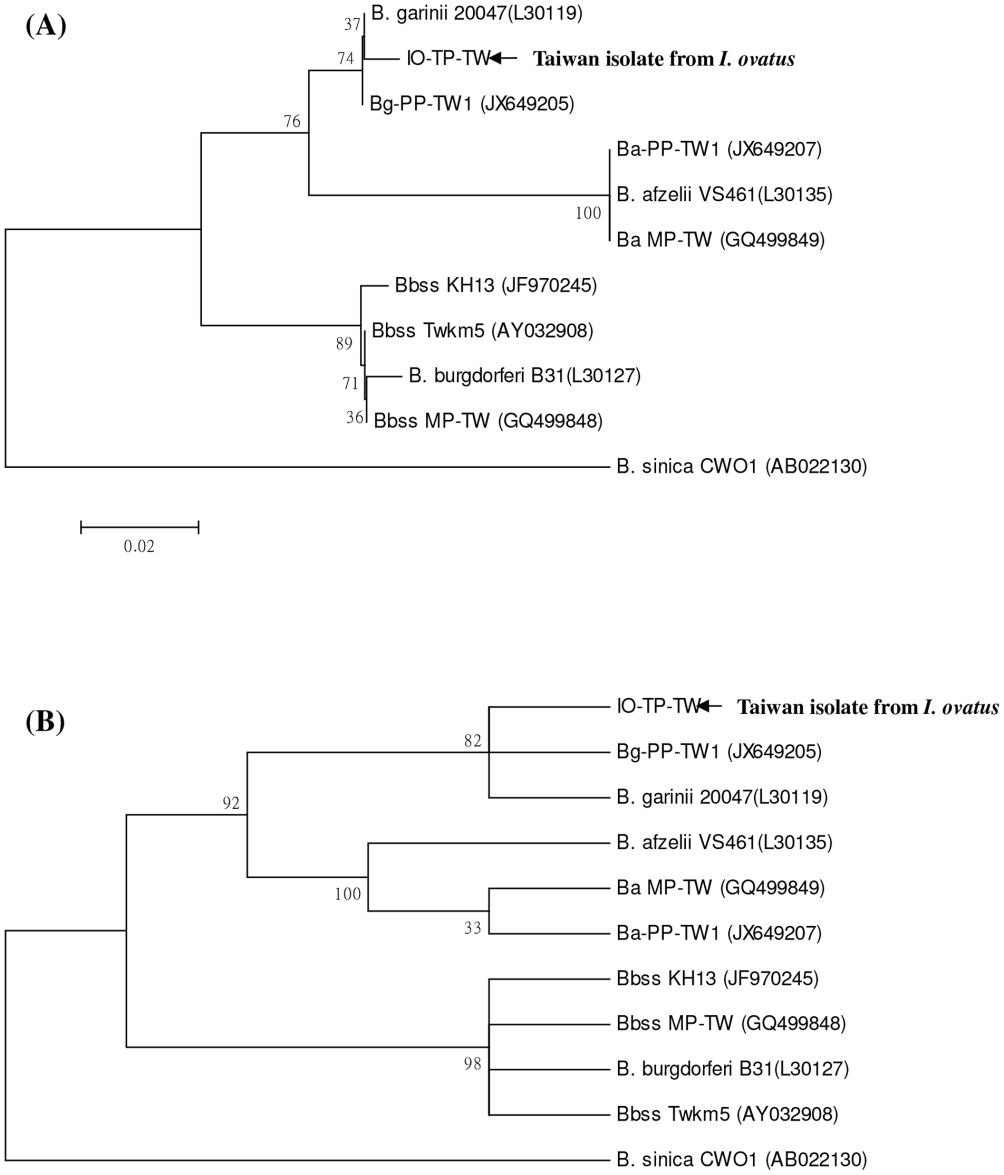
Phylogenetic relationships based on the 5S (*rrf*) –23S (*rrl*) rRNA gene sequences of *Borrelia* spirochetes isolated from *I. ovatus* tick (strain IO-TP-TW) and other biological sources (i.e., human, tick and rodent) in Taiwan. The trees were constructed and analyzed by (**A**) neighbour-joining and (**B**) maximum parsimony methods using 1000 bootstraps replicates. Numbers at the nodes indicate the percentages of reliability of each branch of the tree. Branch lengths are drawn proportional to the estimated sequence divergence.

To clarify the genetic identity of *B. burgdorferi*-like spirochetes, we employed nested PCR assays to achieve a high sensitivity and specificity for *Borrelia* detection. In this study, nested PCR assays reveal amplified DNA fragments of approximately 250 bp (*rrf-rrl* gene) and 320 bp (*ospA* gene) from spirochetes isolated from the dissected tissue of an *I. ovatus* tick. Phylogenetic analyses based on *rrf-rrl* and *ospA* genes also reveal that this detected spirochete represent one monophyletic group closely affiliated to the genospecies of *B. garinii*, and can be distinguished clearly from other *Borrelia* genospecies by both neighbour-joining and maximum parsimony methods (Figs. 3 and 4). Based on comparison of 5S–23S intergenic spacer amplicon gene sequences among 21 *Borrelia* spirochetes, the genetic identity of strain IO-TP-TW isolated from an *I. ovatus* tick of Taiwan was verified with a high sequence homology (96.7 to 98.1% similarity) within the genospecies of *B. garinii* (Table 2) and that can be distinguished from other genospecies of *Borrelia* spirochetes. Interspecies analysis based on the genetic distance values indicates a lower level (0.005 to 0.022) of genetic divergence (GD) within the genospecies of *B. garinii*, and strain IO-TP-TW isolated from an *I. ovatus* tick of Taiwan was genetically more distant (GD >0.113) to the strains identified in *I*. *ovatus* collected from Japan and China (Table 3). Intraspecies analysis also reveals a higher homogeneity (GD<0.005) between tick (strain IO-TP-TW) and human (strain Bg-PP-TW1) isolate of *B. garinii* in Taiwan (Table 3). In addition, phylogenetic analysis of *Borrelia* spirochetes isolated from various biological sources (i.e., human, tick, and rodent) of Taiwan also reveals a genetic affiliation between the strains detected in *I*. *ovatus* tick (strain IO-TP-TW) and in human skin (strain Bg-PP-TW1) (Fig. 5).

**Table 2:** Sequence similarity among 5S (*rrf*) –23S (*rrl*) gene sequences of *Borrelia* strains detected in *Ixodes ovatus* tick from cat, other sources in Taiwan, and strains of other genospecies of *Borrelia*.

Genospecies and strain^a^	B31	*Bb*ss-MP-TW	Twkm5	KH13	20047	IO-TP-TW	*Bg*-PP-TW1	NT29	CT7p	VS461	*Ba*-MP-TW	*Ba*-PP-TW1	VS116	HO14	CWO1
*Bb*ss B31	-	99.5	99.5	97.6	92.4	90.5	91.9	91.9	91.9	85.7	85.7	85.2	89.0	80.0	81.0
*Bb*ss-MP-TW		-	100	98.1	92.9	91.0	92.4	92.4	92.4	86.2	86.2	85.7	89.5	80.5	81.4
Twkm5			-	98.1	92.9	91.0	92.4	92.4	92.4	86.2	86.2	85.7	89.5	80.5	81.4
KH13				-	91.0	91.9	90.5	90.5	90.5	84.3	84.3	83.8	87.6	79.5	80.5
*Bg* 20047					-	98.1	99.5	99.6	98.6	89.0	89.0	88.6	92.4	82.4	81.9
IO-TP-TW						-	97.6	96.7	96.7	87.1	87.1	86.7	90.5	80.5	80.0
* Bg*-PP-TW1							-	98.1	98.1	88.6	88.6	89.0	91.9	82.4	81.4
NT29								-	100	87.6	87.6	87.1	91.9	81.9	82.4
CT7p									-	87.6	87.6	87.1	91.9	81.9	82.4
*Ba* VS461										-	100	99.5	86.7	89.5	82.9
*Ba*-MP-TW											-	99.5	86.7	89.5	82.9
*Ba*-PP-TW1												-	86.2	89.5	82.4
*Bv* VS116													-	81.9	81.0
*Bj* HO14														-	87.6
*Bs* CWO1															-

a Strains: B31, *Bbss*-MP-TW, Twkm5 and KH13, *B. burgdorferi* sensu stricto (*Bbss*); 20047, IO-TP-TW, *Bg*-PP-TW1, NT29 and CT7p, *B. garinii* (*Bg*); VS461, *Ba*-MP-TW and *Ba*-PP-TW1, *B. afzelii* (*Ba*); VS116, *B. valaisiana* (*Bv*); HO14, *B. japonica* (*Bj*); CWO1, *B. sinica* (*Bs*).

**Table 3:** Inter- and Intra-species analysis of genetic distance values^a^ based on the 5S (*rrf*) –23S (*rrl*) gene sequences among *Borrelia* strains detected in *Ixodes ovatus* tick from cat, other sources in Taiwan, and strains of other genospecies of *Borrelia*.

Genospecies and strain^b^	B31	*Bb*ss-MP-TW	Twkm5	KH13	20047	IO-TP-TW	*Bg*-PP-TW1	NT29	CT7p	VS461	*Ba*-MP-TW	*Ba*-PP-TW1	VS116	HO14	CWO1
*Bb*ss B31	-														
*Bb*ss-MP-TW	0.005	-													
Twkm5	0.005	0.000	-												
KH13	0.011	0.005	0.005	-											
*Bg* 20047	0.071	0.065	0.065	0.071	-										
IO-TP-TW	0.078	0.071	0.071	0.078	0.005	-									
*Bg*-PP-TW1	0.071	0.065	0.065	0.071	0.000	0.005	-								
NT29	0.078	0.071	0.071	0.078	0.016	0.022	0.016	-							
CT7p	0.078	0.071	0.071	0.078	0.016	0.022	0.016	0.000	-						
*Ba* VS461	0.107	0.100	0.100	0.107	0.067	0.073	0.067	0.086	0.086	-					
*Ba*-MP-TW	0.107	0.100	0.100	0.107	0.067	0.073	0.067	0.086	0.086	0.000	-				
*Ba*-PP-TW1	0.107	0.100	0.100	0.107	0.067	0.073	0.067	0.086	0.086	0.000	0.000	-			
*Bv* VS116	0.105	0.098	0.098	0.105	0.072	0.078	0.072	0.078	0.078	0.108	0.108	0.018	-		
*Bj* HO14	0.150	0.142	0.142	0.135	0.106	0.113	0.106	0.113	0.113	0.065	0.065	0.065	0.114	-	
*Bs* CWO1	0.171	0.163	0.163	0.154	0.143	0.151	0.143	0.135	0.135	0.205	0.205	0.205	0.152	0.051	-

a The pairwise distance calculation was performed by the method of Kimura 2-parameter, as implemented in MEGA 4 (Tamura et al., 2007).

b Strains: B31, *Bbss*-MP-TW, Twkm5 and KH13, *B. burgdorferi* sensu stricto (*Bbss*); 20047, IO-TP-TW, *Bg*-PP-TW1, NT29 and CT7p, *B. garinii* (*Bg*); VS461,

*Ba*-MP-TW and *Ba*-PP-TW1, *B. afzelii* (*Ba*); VS116, *B. valaisiana* (*Bv*); HO14, *B. japonica* (*Bj*); CWO1, *B. sinica* (*Bs*).

## Discussion

This report provides the first convincing evidence of *B. garinii* spirochete isolated and identified from an adult *I*. *ovatus* tick ectoparasitized on stray cat in Taiwan. In our previous investigations, *Borrelia* spirochetes were isolated from various rodent hosts captured at various locations in Taiwan [24] and the *I. granulatus* tick was recognized as the principle vector tick for enzootic transmission of *B. burgdorferi*-like spirochetes in Taiwan [29]. In addition to the diagnosis of human borreliosis in Taiwan [26], [27], [54], [55], the identification of *Borrelia* spirochetes within possible vector ticks is required to clarify the natural transmission cycle as well as the risk of human infection in Taiwan. Indeed, results from the present study confirm the existence of *B. burgdorferi*-like spirochetes in an adult *I. ovatus* tick and reveal that this detected spirochete is genetically affiliated to the genospecies of *B. garinii* (Table 3 and Figs. 3 and 4). Further investigations focusing on the seasonal abundance of *I. ovatus* ticks and the prevalence of spirochetal infection among *I. ovatus* ticks collected from stray and domestic cats would help to illustrate the ecologic feature of *I. ovatus* and the possibility for transmission of Lyme spirochetes to human by *I. ovatus* tick in Taiwan.

The ability of *I*. *ovatus* tick to serve as a vector responsible for the transmission of Lyme spirochetes to humans remains elusive. Although *I. ovatus* has been recognized as the common tick species ectoparasitized on humans in various countries of Asia [15]–[19], no human cases of *Borrelia* infection transmitted by *I. ovatus* ticks have been confirmed. Previous investigations demonstrated that the field-collected adult *I. ovatus* ticks were highly infected with *Borrelia* spirochetes and these spirochetes found within *I. ovatus* ticks were thought to be a new species that are distinct from the *Borrelia* species associated with human Lyme disease [3], [21]–[23]. However, it is assumed that different genospecies of *Borrelia* spirochetes can be associated with distinct or same vector tick throughout the world [2], [44]. Indeed, results from the previous studies indicate that different genospecies of *Borrelia* spirochetes (i.e., *B. burgdorferi* sensu stricto, *B. valaisiana* and *B. Yangtze*) have been isolated or detected from *I. granulatus* ticks collected from various countries of Asia [13], [28], [29], [56]. In addition to the spirochetal agents of *B. japonica* and *B. sinica* identified in *I. ovatus* ticks of Japan and China, results from this study also verify the existence of *B. garinii* (a species associated with human Lyme disease) in an *I. ovatus* tick removed from stray cat in Taiwan. This finding revealed the genetic diversity of *Borrelia* spirochetes discovered in *I. ovatus* ticks in Asia. However, there is no direct link between this species of *B. garinii* and the vector capability of *I. ovatus*. Accordingly, the vector competence of *I. ovatus* tick for the transmission of *Borrelia* spirochetes to humans needs to be further determined.

The pathogenecity to humans of *B. garinii* isolated from *I. ovatus* tick in Taiwan remains undetermined. Although *B. garinii* has been recognized as the predominant *Borrelia* species detected in field-collected *I. ricinus* and *I. persulcatus* ticks [10], [39], [56], [57], the *B. garinii*-related spirochetes has never been isolated from *I. ovatus* tick. Indeed, all the spirochetal isolates from *I. ovatus* ticks in Asia were identified as new species that are not associated with the spirochetal isolates from humans [3], [21]–[23]. In a laboratory experiment, the susceptibility of *I. ovatus* tick for the transmission of human borreliosis was analyzed by xenodiagnosis and results indicated the insusceptible of *I. ovatus* to human-derived spirochetes [58]. However, the host-associated selection of genetic diversity of *Borrelia* species was proposed [59] and enzoonotic transmission by tick species that rarely feed on humans had also been suggested as the possible factors responsible for the under estimation of human cases [60]. Indeed, a total of thirteen predominant *Borrelia* genospecies have been identified and only three genospecies (i.e., *B. burgdorferi* sensu stricto, *B. garinii*, and *B. afzelii*) are highly pathogenic to humans [44]. Thus, the pathogenecity of *I. ovatus*-derived *B. garinii* to cause human infection in Asia is still ambiguous and needs further clarification.

Because of the unique genomic character with two tandemly duplicated copies of 5S (*rrf*) –23S (*rrl*) intergenic spacer genes existed in *B. burgdorferi* spirochetes [35], [36], the genetic identity of *B. burgdorferi* spirochetes can be clarified by their differential reactivities with genospecies-specific PCR primers targeting the 5S (*rrf*) –23S (*rrl*) intergenic spacer amplicon gene. Indeed, genetic heterogeneity can be further classified among *B. burgdorferi* isolates that were previously identified as the same genospecies of atypical strains of *Borrelia* spirochetes [38], [39]. Results from the present study also verify that the genetic identity of *Borrelia* spirochete detected within an *I. ovatus* tick of Taiwan is highly homogeneous within the genospecies of *B. garinii*, and was clearly distinguished from other genospecies of *Borrelia* spirochetes (Table 2). Interspecies analysis based on the genetic distance (GD) values reveal that *B. garinii* (strain IO-TP-TW) isolated from an *I. ovatus* tick of Taiwan was genetically more distant (GD >0.113) to the strains (i.e., *B. japonica* and *B. sinica*) identified in *I*. *ovatus* collected from Japan and China (Table 3). Intraspecies analysis also reveals a higher homogeneity (GD<0.005) between tick (strain IO-TP-TW) and human (strain Bg-PP-TW1) isolates of *B. garinii* in Taiwan (Table 3). These observations demonstrate the higher association between *I. ovatus*–derived and human–derived isolates of *B. garinii* in Taiwan. Further application of these genospecies-specific PCR tools to analyze the 5S (*rrf*) –23S (*rrl*) genes of *Borrelia* spirochetes detected in various tick species and reservoir hosts would help to clarify the genetic divergence of *Borrelia* spirochetes transmitted in the natural cycle of Taiwan.

Phylogenetic relationships among *Borrelia* spirochetes can be determined by analyzing their sequence homogeneity of the 5S–23S intergenic spacer amplicon (*rrf-rrl*) and outer surface protein A (*ospA*) genes. Indeed, the sequence analysis of 5S (*rrf*) –23S (*rrl*) intergenic spacer amplicon and *ospA* genes among various *Borrelia* spirochetes had been proved useful to evaluate the taxonomic relatedness of *Borrelia* spirochetes derived from various biological and geographical sources [26], [27], [31], [37], [40], [41]. Although PCR amplification of the intergenic spacer region located between the *rrf* and *rrl* genes of *B. burgdorferi* sensu lato had been reported to generate a DNA fragment of approximately 226–266 bp long [48], the variation of nucleotide sequence depends on the diversity of the strain or genospecies of *Borrelia* spirochetes, and may actually represent the genetic distance of phylogenetic divergence between or within the genospecies of *Borrelia* spirochetes [2], [5], [48]. In this study, phylogenetic analysis based on the sequences of 5S (*rrf*) –23S (*rrl*) intergenic spacer amplicon and *ospA* genes of *Borrelia* spirochetes isolated from an *I. ovatus* of Taiwan and other biological sources demonstrated a high sequence homogeneity among *Borrelia* strains within the genospecies of *B. garinii* and a high genetic heterogeneity among different genospecies of *Borrelia* strains (Figs. 3 and 4). The phylogenetic trees constructed by either NJ or MP analysis strongly support the discrimination recognizing the separation of different lineages of *Borrelia* species detected from various biological and geographical sources. In addition, intraspecies analysis of *Borrelia* species detected in various sources (i.e., tick, rodent and human isolates) from Taiwan demonstrates a higher genetic affiliation between the tick (strain IO-TP-TW) and human (Bg-PP-TW1) isolates of *B. garinii* (Fig. 5). Accordingly, these observations may imply the risk of human infection transmitted by *I. ovatus*.

## Conclusions

Our report provides the first evidence regarding the existence of *B. garinii* spirochetes within *I. ovatus* tick collected from stray cat in Taiwan. The genetic identity of this detected spirochete (strain IO-TP-TW) reveals a high sequence homology associated with human isolate (strain Bg-PP-TW1) of *B. garinii* in Taiwan. Because of the close contact of cats with humans and their ability to serve as carriers for vector ticks, the information regarding the tick species ectoparasitized on cats and the prevalence of spirochetal infection in infested ticks is pre-requisite for the prevention of tick-borne human infections. Further investigations focusing on the detection of *Borrelia* spirochetes within different vector ticks ectoparasitized on stray and domestic cats would help to clarify the significance of genetic diversity of *Borrelia* spirochetes in relation to the epidemiological features of Lyme disease infection in Taiwan.
